# Aryl hydrocarbon receptor blocks aging-induced senescence in the liver and fibroblast cells

**DOI:** 10.18632/aging.204103

**Published:** 2022-05-26

**Authors:** Ana Nacarino-Palma, Eva M. Rico-Leo, Judith Campisi, Arvind Ramanathan, Francisco J. González-Rico, Claudia M. Rejano-Gordillo, Ana Ordiales-Talavero, Jaime M. Merino, Pedro M. Fernández-Salguero

**Affiliations:** 1 Departamento de Bioquímica y Biología Molecular, Facultad de Ciencias, Universidad de Extremadura, Badajoz 06071, Spain; 2 Instituto Universitario de Investigación Biosanitaria de Extremadura (INUBE), Badajoz 06071, Spain; 3 Buck Institute for Research on Aging, Novato, CA 94945, USA; 4 Lawrence Berkeley National Laboratory, Berkeley, CA 94720, USA

**Keywords:** aryl hydrocarbon receptor, hepatocarcinogenesis, senescence, metabolism

## Abstract

Aging impairs organismal homeostasis leading to multiple pathologies. Yet, the mechanisms and molecular intermediates involved are largely unknown. Here, we report that aged aryl hydrocarbon receptor-null mice (*AhR−/−*) had exacerbated cellular senescence and more liver progenitor cells. Senescence-associated markers β-galactosidase (SA-β-Gal), p16^Ink4a^ and p21^Cip1^ and genes encoding senescence-associated secretory phenotype (SASP) factors TNF and IL1 were overexpressed in aged *AhR−/−* livers. Chromatin immunoprecipitation showed that AhR binding to those gene promoters repressed their expression, thus adjusting physiological levels in *AhR+/+* livers. MCP-2, MMP12 and FGF secreted by senescent cells were overproduced in aged AhR-null livers. Supporting the relationship between senescence and stemness, liver progenitor cells were overrepresented in *AhR−/−* mice, probably contributing to increased hepatocarcinoma burden. These AhR roles are not liver-specific since adult and embryonic AhR-null fibroblasts underwent senescence in culture, overexpressing SA-β-Gal, p16^Ink4a^ and p21^Cip1^. Notably, depletion of senescent cells with the senolytic agent navitoclax restored expression of senescent markers in *AhR−/−* fibroblasts, whereas senescence induction by palbociclib induced an AhR-null-like phenotype in *AhR+/+* fibroblasts. AhR levels were downregulated by senescence in mouse lungs but restored upon depletion of p16^Ink4a^-expressing senescent cells. Thus, AhR restricts age-induced senescence associated to a differentiated phenotype eventually inducing resistance to liver tumorigenesis.

## INTRODUCTION

The aryl hydrocarbon receptor (AhR) is a transcription factor with important roles in toxicology and cell physiology [1–3]. AhR has a significant role in essential signaling pathways controlling the cell cycle, cell-cell contact, apoptosis, angiogenesis, differentiation and pluripotency [4–7]. Deregulation of these pathways, critical for maintaining homeostasis, predispose organisms to develop several pathologies, including cancer [1, 8, 9].

Recent findings uncovered important roles for AhR in cell differentiation and pluripotency, supporting its potential as therapeutic target [5, 8, 10]. Our group demonstrated that AhR adjusts liver and lung regeneration upon injury by controlling the proliferation and expansion of pluripotent stem-like cells [11, 12]. Accordingly, lack of AhR (*AhR−/−*) induces an undifferentiated and non-polyploid phenotype in the mouse liver, which persists from preweaning to adulthood [13]. Moreover, increased numbers of stem cells in *AhR−/−* mouse livers correlate with their higher susceptibility to develop diethylnitrosamine (DEN)-induced hepatocarcinomas [1, 12].

In addition to multiple genetic and environmental causes, aging remains a major risk factor for developing cancer [14]. During aging, biological systems undergo progressive degeneration and accumulate molecular changes that compromise and decrease physiological and fertility functions, ultimately leading to loss of homeostasis and age-related pathologies. In addition, impaired cellular and molecular functions during aging may trigger new and aberrant regenerative capacities leading to hyperplasic pathologies [15].

Most age-related pathologies, degenerative or hyperplastic, are linked to a stress response called senescence. Senescence consists of an essentially irreversible cell cycle arrest, changes in chromatin organization and altered gene expression patterns [16, 17]. The latter includes secretion of pro-inflammatory cytokines, chemokines, growth factors and proteases, generating a senescence-associated secretory phenotype (SASP) [18]. Senescence halts the proliferation of damaged cells, and thus protects against the development of cancer [15, 19, 20]. Moreover, the fact that SASP has multiple paracrine activities suggests that senescence also promotes tissue repair and regeneration upon injury [20–22]. Conversely, SASP factors can enhance tumorigenesis by promoting proliferation, metastasis and immunosuppression [23]. A relevant link has been identified between senescence and cellular reprogramming since *in vivo* reprogramming can induce tumors [24–27] and aging-associated senescence [22] by mechanisms probably requiring an exacerbated inflammatory status.

An early report revealed that mouse embryo fibroblasts (MEFs) from *AhR−/−* mice reached senescence earlier than *AhR+/+* cells during adipogenic differentiation [28]. Later work showed that AhR reduces smoking-induced inflammation in the lung parenchyma through, among other mechanisms, the control of senescence [29]. In addition, air-born particles can promote cellular senescence through AhR by producing reactive oxygen species (ROS), causing epigenetic modifications leading to p16^Ink4a^ activation [30].

Here, we report that aged mice lacking AhR expression have a significant increase in hepatic senescence that correlates with increased liver progenitor cells and a higher incidence of hepatocarcinoma. AhR deficiency also induced senescence in adult and embryonic fibroblasts, which could be modulated by senolytics or senescence promoters in a receptor-dependent manner. Given the recently discovered link between senescence and reprogramming, it seems plausible that AhR acts to properly control the balance of both processes during physiological aging. This relationship may explain the exacerbated regenerative response of AhR-null mice after liver and lung injury.

## RESULTS

### AhR depletion increases liver tumor burden with aging

**Figure 1:**
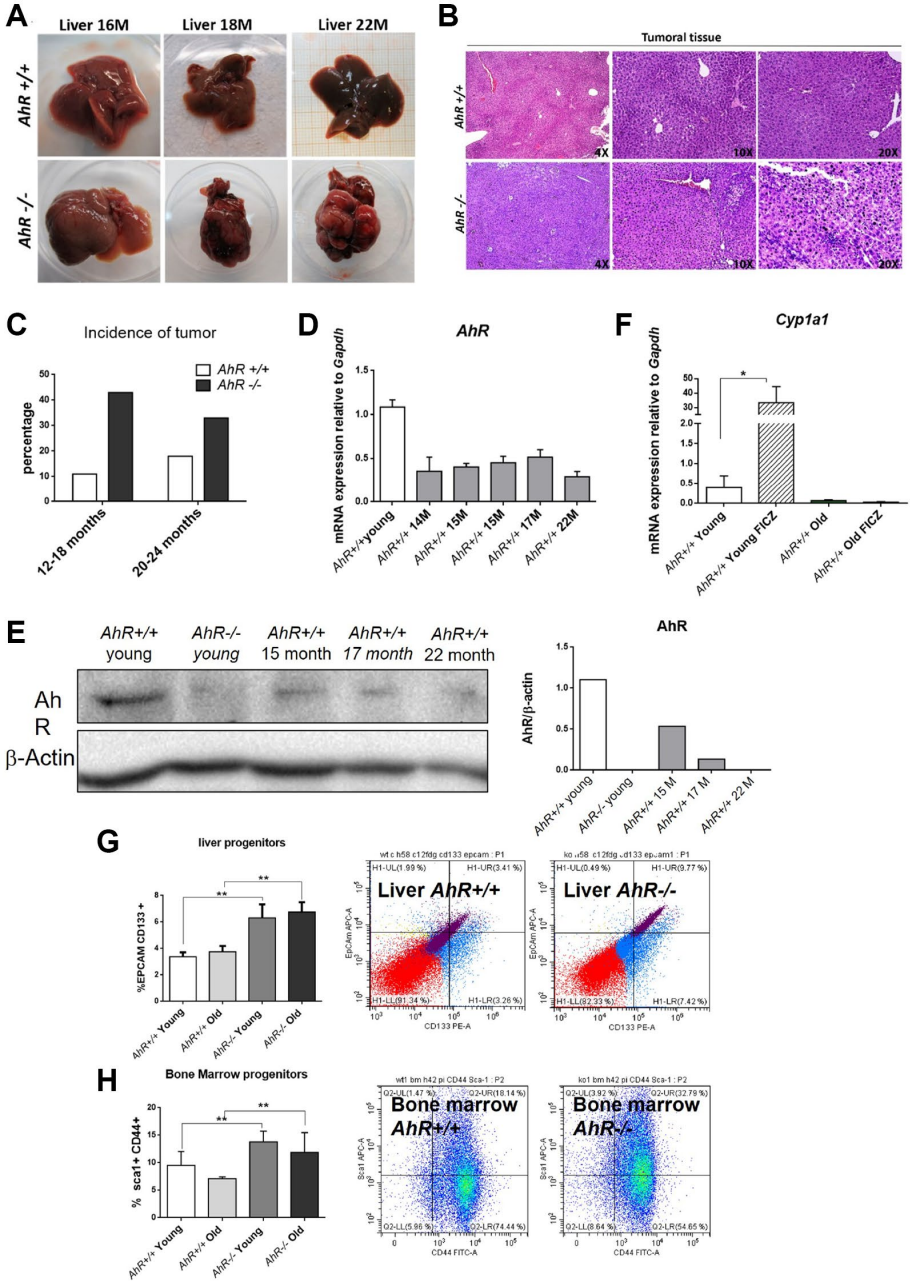
**AhR depletion increases liver tumorigenesis with aging.** (**A**) Representative tumors developed by *AhR+/+* and *AhR−/−* at the indicated ages. (**B**) Haematoxylin and Eosin staining of liver tumor sections from *AhR+/+* and *AhR−/−* mice at 22 months of age. Note the abundance of pycnotic nuclei in *AhR−/−* tumors. (**C**) Quantification of the number of liver tumors in mice of both genotypes at two age intervals. (**D**) AhR mRNA levels in *AhR+/+* livers at the indicated ages using RT-qPCR and the oligonucleotides indicated in Supplementary Table 1. (**E**) AhR protein levels were analyzed in liver extracts at the indicated ages by immunoblotting. β-Actin was used to normalize protein levels. (**F**) *AhR+/+* mice were injected i.p. with 4 mg/kg FICZ and mRNA levels of the AhR canonical target gene *Cyp1a1* were determined by RT-qPCR using the oligonucleotides indicated in Supplementary Table 1. (**G**) Liver progenitor cells were analyzed by FACS using antibodies against CD133-PE and EPCAM-APC. Distribution of cell subpopulations and gating from representative experiments are shown. (**H**) Bone marrow progenitor cells were analyzed by FACS using the markers CD44-FITC and Sca1-APC. Distribution of cell subpopulations and gating from representative experiments are shown. *Gapdh* was used to normalize target gene expression (△Ct) and 2^−△△Ct^ to calculate changes in mRNA levels with respect to wild type or untreated conditions. Data are shown as mean + SD (^*^P < 0.05; ^**^P < 0.01).

Adult AhR-null mice maintain an undifferentiated and non-polyploid liver phenotype [13] that could favor age-related hepatocarcinogenesis. To address this possibility, we examined *AhR+/+* and *AhR−/−* mice aged 15 to 22 months for the presence of liver tumors. Lack of AhR led to liver tumors that were evident at 18 months of age and accounted for a large fraction of the liver at 22 months (Figure 1A). AhR−/− tumors were larger in size, and had pyknotic nuclei, suggestive of chromatin condensation and replicative blockade (Figure 1B). Tumor incidence reached close to 45% in AhR-null mice but remained below 20% in AhR wild type animals (Figure 1C). Additionally, spontaneous hepatocarcinogenesis affected close to 80% of *AhR−/−* mice in the C57BL/J background, confirming that AhR depletion favors liver tumorigenesis (data not shown and [12]).

We next analyzed AhR expression during aging. Figure 1D, shows that AhR mRNA levels drastically decline in *AhR+/+* 14-month-old livers and remain low until at least 22 months of age. A similar expression profile was found by immunoblotting analysis of liver extracts from both genotypes (Figure 1E). Treatment with the physiological agonist FICZ significantly increased liver mRNA levels of the canonical target gene *Cyp1a1* in young *AhR+/+* mice. Further, aging markedly reduced the ability of FICZ to induce *Cyp1a1* expression in AhR-expressing liver (Figure 1F), supporting the hypothesis that AhR downregulation has a causal role in liver hepatocarcinogenesis [12, 13]. In agreement with the known role of AhR in controlling pluripotency and stemness [8, 10], enhanced hepatocarcinogenesis in AhR-null liver correlated with their higher numbers of liver progenitor/stem-like cells in both young and aged animals (Figure 1G). Interestingly, AhR deficiency also increased bone marrow progenitor cells regardless of age (Figure 1H), thus confirming the role of this receptor in maintaining the population of undifferentiated cells in different organs. Enhanced tumor burden in *AhR−/−* mouse liver also correlated with increased expression of the glucose transporter Glut4 in both old and young mice (Supplementary Figure 1A). Accordingly, glucose uptake, as determined by the level of hexokinase activity, was significantly higher in old AhR-null compared to *AhR+/+* mice (Supplementary Figure 1B). Thus, aging in *AhR−/−* mice induces preferential glycolytic metabolism that could fuel their greater susceptibility to develop liver cancer and a Warburg effect.

### AhR deficient livers have increased senescence with aging

**Figure 2:**
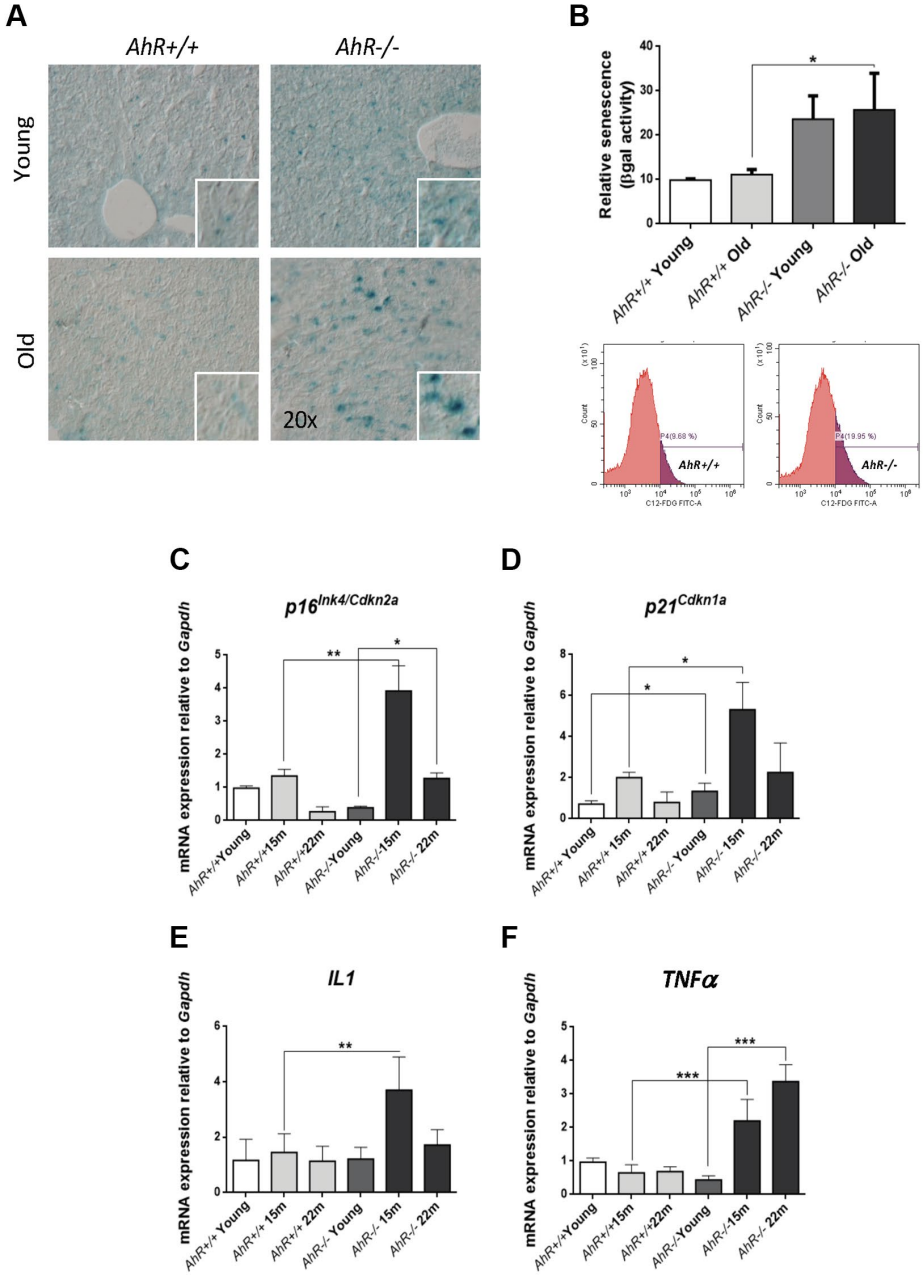
**Cell senescence increases with age in AhR-deficient liver.** (**A**) SA-β-Gal activity was analyzed in *AhR+/+* and *AhR−/−* liver sections by staining with the chromogenic substrate X-Gal. (**B**) SA-β-Gal activity was analyzed by FACS in isolated liver cells (gentleMACS) using the fluorescent substrate C12FDG. (**C**–**F**) mRNA levels of senescence driver genes *p16^Ink4a^* (**C**) and *p21^Cip1^* (**D**) and SASP-related genes IL1 (**E**) and TNFα (**F**) were analyzed by RT-qPCR in *AhR+/+* and *AhR−/−* livers at the indicated ages. Oligonucleotides used are indicated in Supplementary Table 1. *Gapdh* was used to normalize target gene expression (△Ct) and 2^−△△Ct^ to calculate changes in mRNA levels with respect to wild type or untreated conditions. Data are shown as mean + SD (^*^P < 0.05; ^**^P < 0.01; ^***^P < 0.001).

Undifferentiated status favors hepatocarcinogenesis [31, 32], whereas senescence can promote reprogramming in aged undifferentiated tumors [22, 33]. We investigated whether AhR deficiency in aged livers could induce senescence and eventual hepatic tumors. Senescence-associated β-galactosidase activity (SA-β-Gal) increased markedly in liver sections from aged *AhR−/−* mice compared to *AhR+/+* counterparts (Figure 2A). Quantification of senescence using C12FDG-labelled hepatic cells and FACS confirmed that AhR deficiency significantly expanded the number of senescent cells upon aging (Figure 2B). We also used RT-qPCR to quantify liver mRNA levels of genes considered drivers of senescence. *p16^Ink4a^* mRNA levels markedly increased in *AhR−/−* livers at 15 months of age, then decreased to lower levels at 22 months of age (Figure 2C). Similarly, *p21^Cip1^* transiently increased in *AhR−/−* livers at 15 months, reaching values significantly higher to those of *AhR+/+* livers; differences in *p21^Cip1^* expression between both genotypes could be seen even in young mice (Figure 2D). The expression of factors that comprise the senescence-associated secretory phenotype (SASP), IL1 and TNFα was also up-regulated with aging in absence of AhR (Figure 2E, 2F), although TNFα remained at higher levels even at the oldest age analyzed (22 months). We detected no significant transcriptional changes between both genotypes with respect to liver p53 or IL6 (data not shown). Thus, AhR limits senescence and carcinogenesis in the liver, which could be related to its potential to maintain differentiation in this organ.

**Figure 3:**
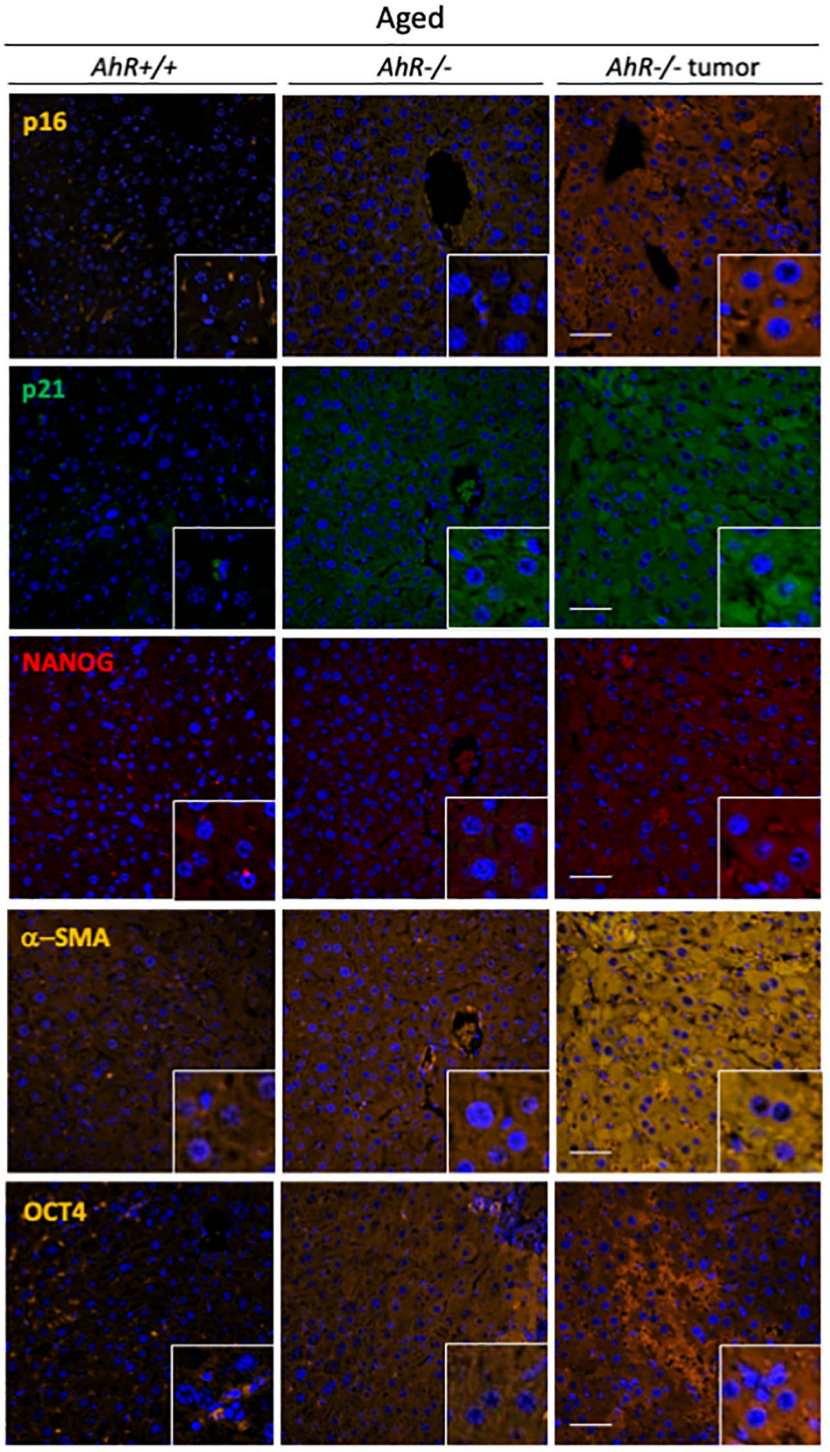
***AhR−/−* livers overexpress senescence and undifferentiation/stemness markers.** Protein levels of senescence markers p16^Ink4^ and p21^Cip1^ and pluripotency/stemness inducers NANOG and OCT4 were analyzed by immunofluorescence in liver sections of aged *AhR+/+* and *AhR−/−* mice and in hepatocarcinomas from *AhR−/−* mice. Expression of α-SMA was also analyzed as indicator of vasculogenesis. Conjugated secondary antibodies labelled with Alexa 488, Alexa 550 and Alexa 633 were used for detection. DAPI staining was used to label cell nuclei. An Olympus FV1000 confocal microscope and the FV10 software (Olympus) were used for the analysis. Scale bar corresponds to 50 μm.

To further correlate undifferentiation and senescence in the liver, we analyzed markers of both differentiation and senescence in non-tumoral tissue from old mice and hepatocarcinoma samples from *AhR+/+* and *AhR−/−* mice by immunofluorescence. Regarding senescence, p16^Ink4a^ and p21^Cip1^were present at higher levels in the liver of aged AhR-null mice, compared to wild mice. Similar staining pattern can be seen in previous works [34, 35] This pluripotency/stemness and undifferentiation inducers NANOG and OCT4 were also overexpressed in *AhR−/−* livers of old mice (Figure 3), in agreement with previous work [36, 37]. Expression of p21^Cip1^ and OCT4 were prevalent with respect to p16^Ink4^ and NANOG, respectively. Well-developed hepatocarcinomas from *AhR−/−* mice (Figure 1) showed even higher levels of these proteins, suggesting that tumorigenesis accentuates the basal undifferentiated and senescent status of aged *AhR−/−* mice. Further, levels of the mesenchymal marker α-smooth muscle actin (α-SMA) increased in hepatocarcinomas and old *AhR−/−* liver compared to livers of *AhR+/+* mice (Figure 3), This result could be related to hepatic phenotype in AhR null mice [38, 39].

**Figure 4:**
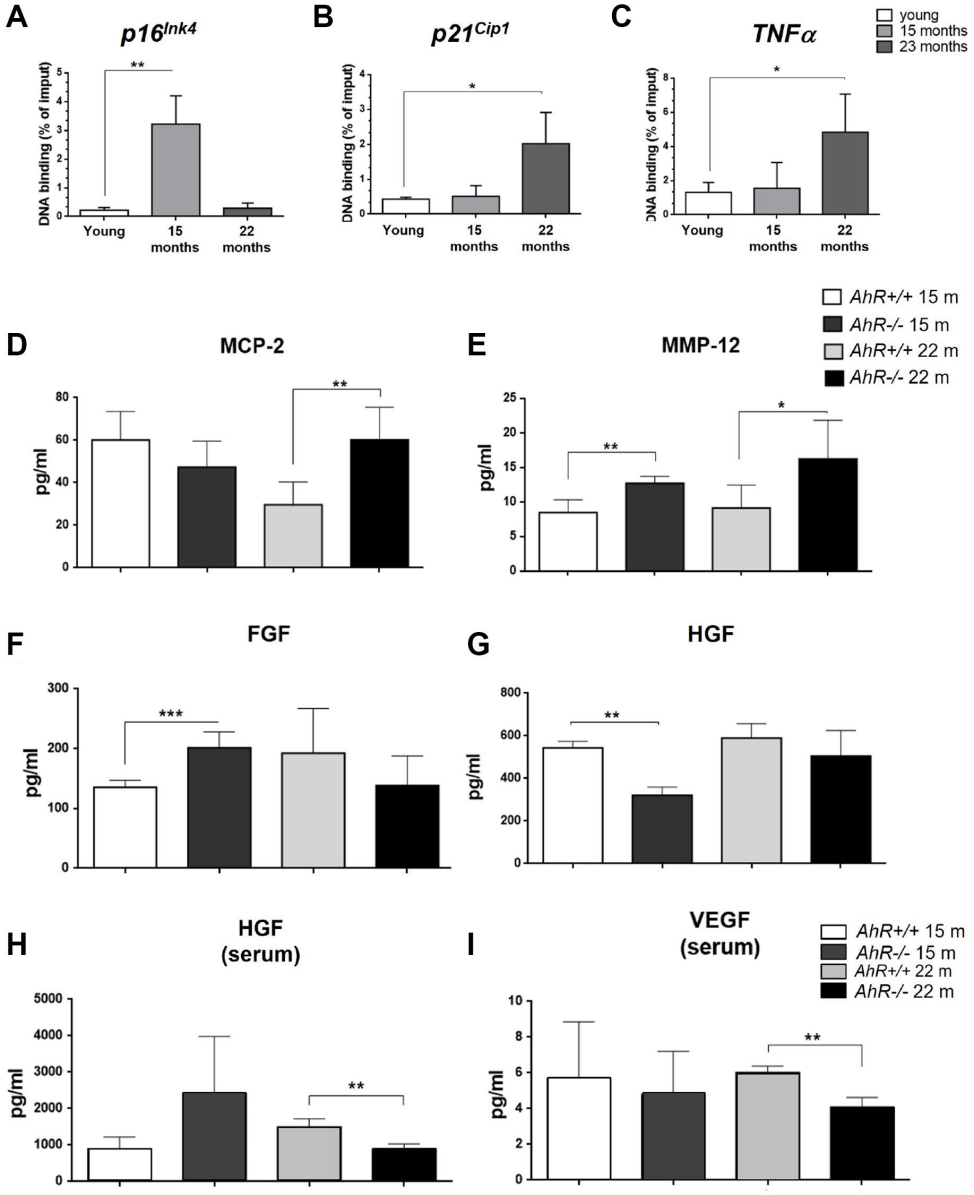
**AhR modulates the senescence-associated secretory phenotype with aging.** (**A**). Chromatin immunoprecipitation (ChIP) for AhR binding to XRE binding sites located in the promoters of *p16^Ink4a^* (**A**), *p21^Cip1^* (**B**) and *TNFα* (**C**). qPCR was used to quantify changes in DNA binding and the results were normalized to the corresponding inputs. Amounts of MCP-2 (**D**), MMP12 (**E**), FGF (**F**) and HGF (**G**) were analyzed in liver homogenates from *AhR+/+* and *AhR−/−* mice at the indicated ages. Levels of HGF (**H**) and VEGF (**I**) VEGF were also determined in sera from mice of the same genotypes and ages. Bio-Plex Multiplex immunoassays kits were used. Oligonucleotides for qPCR are indicated in Supplementary Table 1. *Gapdh* was used to normalize target gene expression (△Ct) and 2^−△△Ct^ to calculate changes in mRNA levels with respect to wild type or untreated conditions. Data are shown as mean + SD (^*^P < 0.05; ^**^P < 0.01; ^***^P < 0.001).

Since senescence markers increased at the mRNA level in absence of AhR (Figure 2), we asked if AhR transcriptionally repressed those genes by direct binding to promoters. Sequence analysis revealed the presence of a AhR-canonical binding site XRE (Xenobiotic response element, 5′-GCGTG-3′) in the promoter region of *p16^Ink4a^, p21^Cip1^* and *TNFα* Chromatin Immunoprecipitation (ChIP) for AhR in liver extracts from young (2 months) and old (15 and 22 months) mice was performed (Figure 4). AhR bound to the *p16^Ink4a^* gene promoter at 15 months but was released in animals at 22 months (Figure 4A). AhR binding to the *p21^Cip1^* and *TNFα* gene promoters was maximal at 22 months of age and nearly basal in younger mice (Figure 4B, 4C). Thus, overexpression of these genes in old *AhR−/−* livers likely results from a lack of transcriptional repression due to an absence of receptor.

### Senescence-associated secretory phenotype factors are regulated in an AhR-dependent manner during liver aging

Factors secreted by senescent cells could contribute to the aging phenotype of *AhR−/−* mouse liver. To explore this possibility, we used immunoassays to analyze the levels of major SASP factors secreted within the liver or into the blood of *AhR+/+* and *AhR−/−* aged mice. In the liver, secretion of MCP2/CCL8, a cytokine that chemoattracts monocytes and macrophages to damaged or tumor areas, was significantly higher in *AhR−/−* mice at 22 months compared to age-matched *AhR+/+* mice (Figure 4D). Matrix metalloproteinase 12 (MMP-12), which stimulates matrix degradation and liver tumor progression, was significantly overexpressed not only in the oldest mice (22 months) but also in mice at the beginning of the aging process (15 months) (Figure 4E). Moreover, fibroblast growth factor (FGF), which seems to inhibit senescence [40], had a trend to decrease in *AhR−/−* liver with aging, despite being at higher levels than wild type mice at 15 months (Figure 4F). Hepatocyte growth factor (HGF), overactivation of which inhibits senescence [41], also declined at 15 months and did not significantly increase in 22 months AhR-null mice (Figure 4G). In serum, HGF levels were significantly lower in 22 months *AhR−/−* mice (Figure 4H), as was the amount of circulating vascular endothelial growth factor (VEGF) (Figure 4I), in agreement with their implication in aging-related endothelial senescence [42]. Thus, the SASP appears to be exacerbated in *AhR−/−* liver during aging.

### Senescence markers are enhanced in adult primary fibroblasts lacking AhR

**Figure 5:**
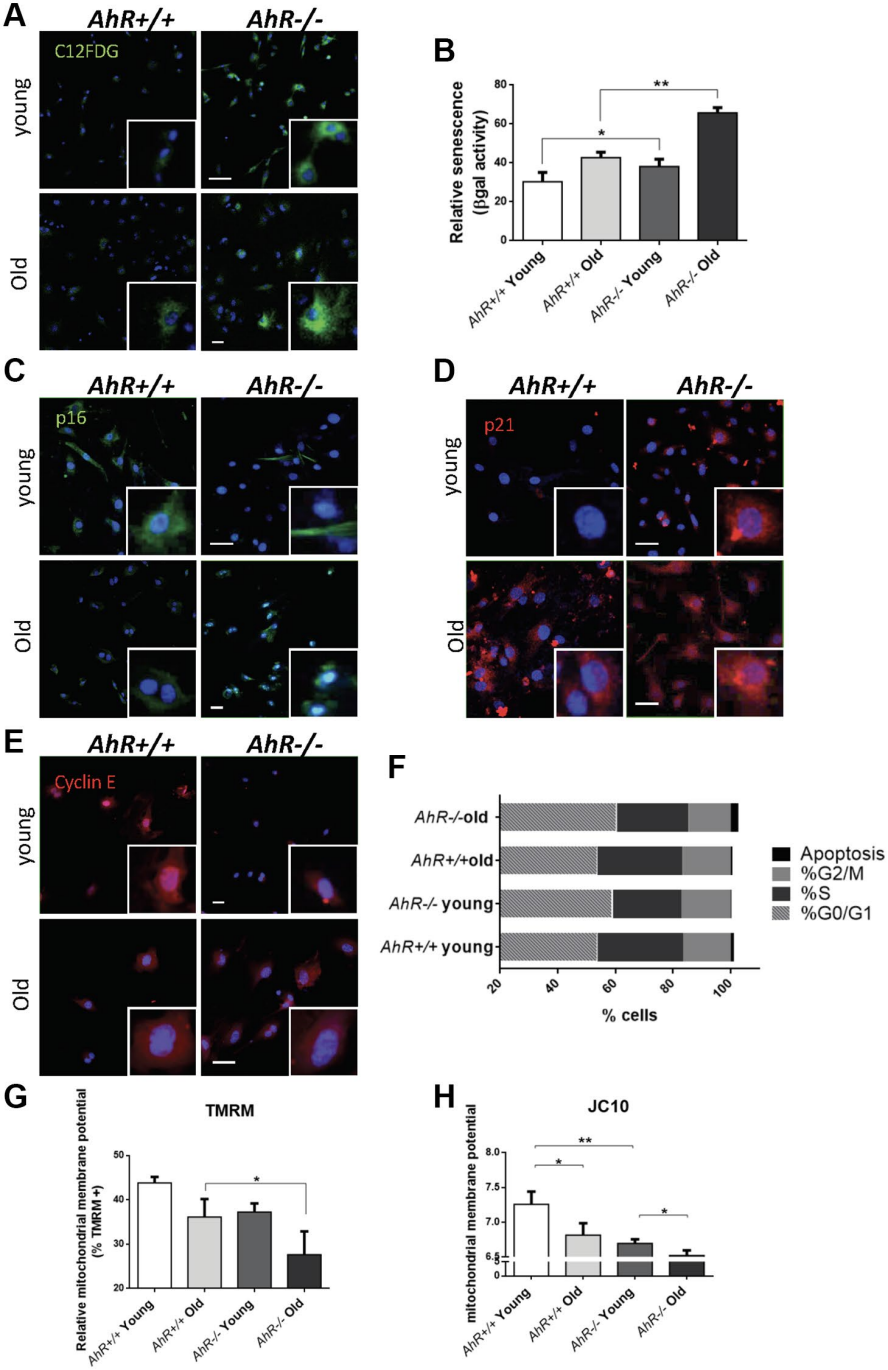
**Senescence increases with aging in adult AhR-null fibroblasts.** (**A**) *AhR+/+* and *AhR−/−* fibroblasts were stained with the SA-β-Gal fluorescent substrate C12FDG to determine senescence levels by confocal microscopy. (**B**) SA-β-Gal activity was also measured by flow cytometry analyzing the percentage of C12FDG positive cells. (**C**–**E**) p16^Ink4a^ (**C**), p21^Cip1^ (**D**) and Cyclin E (**E**) were analyzed by florescence confocal microscopy using specific antibodies in young and aged fibroblast cells. Conjugated secondary antibodies used were Alexa 488 and Alexa 633. DAPI staining was used to label cell nuclei. An Olympus FV1000 confocal microscope with FV10 software (Olympus) were used. (**F**) Percentage in each cell cycle phase. Cell cycle analysis was performed by FACS using propidium iodide staining. (**G**) Mitochondrial membrane potential (MMP) was quantified by the percentage of TMRM positive cells analyzed by Cytoflex S cytometer (Beckman Coulter). (**H**) MMP was also calculated using the JC10 kit for mitochondrial membrane polarization (Sigma-Aldrich). The red/green fluorescence intensity ratio was used to determine MMP activity. Data are shown as mean + SD (^*^P < 0.05; ^**^P < 0.01).

To investigate the role of AhR in senescence in additional cell types, we examined adult primary fibroblasts, which easily undergo senescence [43, 44] and can be readily isolated from *AhR+/+* and *AhR−/−* tissues. We isolated adult tail fibroblasts from young (4–6 weeks) and old (15–22 months) mice of both genotypes, cultured and analyzed for senescence using the SA-β-Gal fluorescent substrate C12FDG (Figure 5). Fluorescent confocal microscopy revealed that both young and old *AhR−/−* TTFs had more senescent cells than *AhR+/+* cells under the same culture conditions (Figure 5A). Fluorescence flow cytometry similarly showed that lack of AhR significantly increased the proportion of senescent cells in *AhR−/−* cells at both ages (Figure 5B). Cells positive for p16^Ink4a^ were equally abundant in young cells of both genotypes but markedly increased in old *AhR−/−* cells (Figure 5C). Regarding p21^Cip1^, a similar response was found in *AhR−/−* cells with aging, showing nuclear expression in a large fraction of cells (Figure 5D). On the other hand, old *AhR−/−* cells had more Cyclin E positive cells than old *AhR+/+* cells (Figure 5E), together with a higher percentage of cells at the G0/G1 boundary that was also present in young AhR-null cells (Figure 5F). These results suggest that a concurrent increase in Cyclin E and tumor suppressors may cause cell cycle blockade, reduced proliferation of adult *AhR−/−* fibroblasts [45, 46] and eventually contribute to higher rates of senescence.

Mitochondrial dysfunction is also indicative of cellular senescence [47]. We measured the mitochondrial membrane potential (MMP) of young and old *AhR+/+* and *AhR−/−* fibroblasts. Flow cytometry using tetramethyl rhodamine (TMRM), a fluorescent cationic dye captured by active mitochondria, showed that old AhR-null cells had less mitochondrial activity than *AhR+/+* cells (Figure 5G). We also used JC10, which aggregates depending on membrane polarization, to support our finding that old *AhR−/−* mice had lower mitochondrial metabolism (Figure 5H).

### Primary fibroblasts lacking AhR expression have increased senescence rates

**Figure 6:**
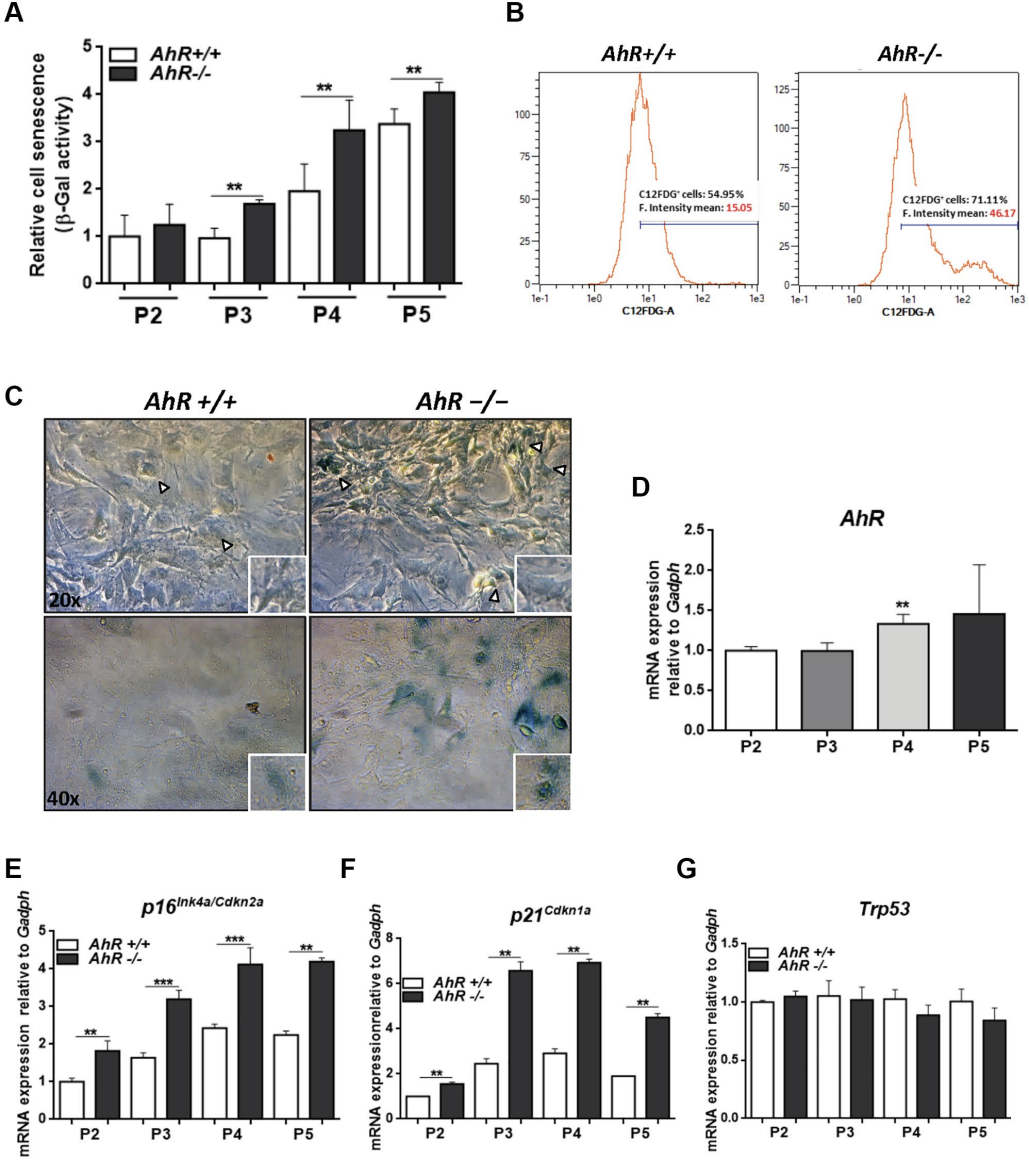
**Lack of AhR enhances *in vitro* cellular senescence in mouse embryonic fibroblasts.** (**A**) Senescence profiles of *AhR+/+* and *AhR−/−* MEFs at the indicated passages as determined by the level of SA-β-gal activity analyzed by FACS using the β-galactosidase fluorescent substrate C12FDG. Results are normalized to wildtype MEFs at passage 2. (**B**) Representative flow cytometric profiles of senescent cells stained with C12FDG in *AhR+/+* and *AhR−/−* MEFs at passage 5. (**C**) SA-β-Gal activity in *AhR+/+* and *AhR−/−* MEFs at passage 5 as determined by staining using X-Gal as substrate. (**D**) *AhR* mRNA expression was determined by RT-qPCR at the indicated cell culture passages. (**E**–**G**) mRNA expression of senescence driver genes *p16^Ink4a^* (**E**)*, p21^Cip1^* (**F**) and *Trp53* (**G**) were determined in *AhR+/+* and *AhR−/−* MEFs by RT-qPCR using the oligonucleotides indicated in Supplementary Table 1. *Gapdh* was used to normalize target gene expression (△Ct) and 2^−△△Ct^ to calculate changes in mRNA levels with respect to wild type or untreated conditions. Data are shown as mean + SD (^**^P < 0.01; ^***^P < 0.001).

AhR-null mouse embryonic fibroblasts (MEF), on passaging in culture, acquire a senescent phenotype earlier than wild type cells [28] and have reduced proliferation potential [48]. Flow cytometry and cytological staining for SA-β-Gal indicated that, upon passage in culture, AhR-null MEFs acquired higher levels of senescence than *AhR+/+* MEFs (significant after passage 3, P3) (Figure 6A–6C). AhR expression increased with increasing passage from P2 to P5 (Figure 6D), suggesting that, in *AhR*+/+ MEFs, AhR up-regulation might restrict senescence and that receptor depletion might exacerbate this process. Consistently, *p16^Ink4a^* and *p21^Cip1^* expression increased in *AhR−/−* MEFs with increasing passage (Figure 6E, 6F). p53 mRNA levels did not significantly change during passage for either genotype (Figure 6G).

**Figure 7:**
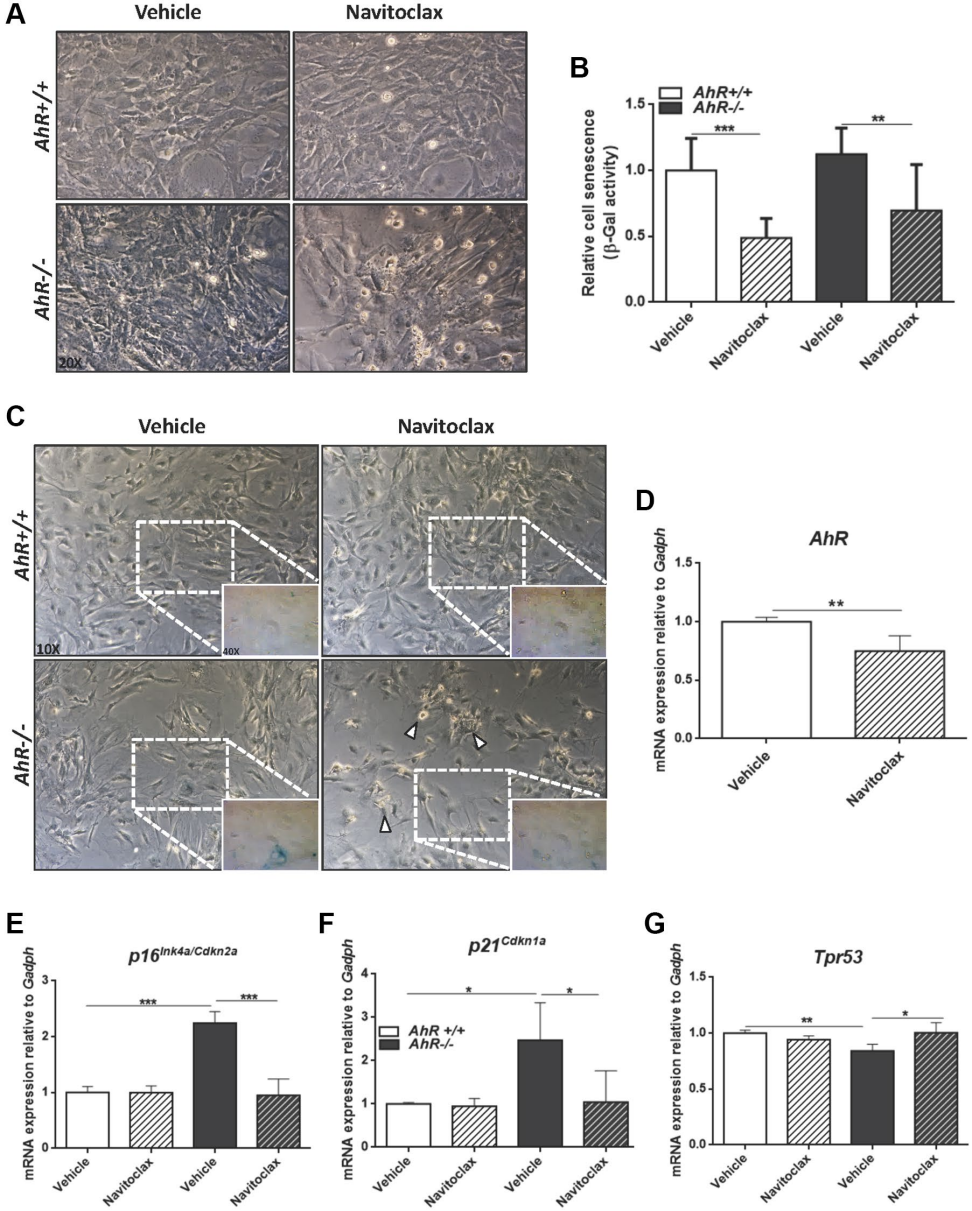
**Senolytic agent Navitoclax restores wild-type mRNA levels of senescence driver genes in *AhR−/−* MEFs.** Embryonic fibroblasts at P4 or P5 were treated with vehicle or 10 μM Navitoclax for 48 h. (**A**) Bright-field microscopy of *AhR+/+* and *AhR−/−* MEFs untreated or treated with Navitoclax. (**B**) Cell senescence measured as percentage of SA-β-Gal activity in MEF cells of both genotypes. SA-β-Gal activity was analyzed by FACS using the β-galactosidase fluorescent substrate C12FDG. Results are normalized to vehicle-treated wild-type MEFs. (**C**) X-Gal staining in untreated and Navitoclax-treated *AhR+/+* and *AhR−/−* MEFs. (**D**) *AhR* mRNA expression was determined by RT-qPCR in both experimental conditions using the oligonucleotides indicated in Supplementary Table 1. (**E**–**G**) mRNA expression of senescence driver genes *p16Ink4a* (**E**), *p21Cip1* (**F**) and *Trp53* (**G**) was determined in *AhR+/+* and *AhR−/−* MEFs by RT-qPCR using oligonucleotides indicated in Supplementary Table 1. *Gapdh* was used to normalize target gene expression (△Ct) and 2^−△△Ct^ to calculate changes in mRNA levels with respect to wild type or untreated conditions. Data are shown as mean + SD (^*^P < 0.05; ^**^P < 0.01; ^***^P < 0.001).

To further characterize the contribution of AhR to cellular senescence, we treated mouse embryo fibroblasts (MEFs) with inhibitors or activators of cell death. Navitoclax is a senolytic that targets BCL2 family member to kill senescent cells [49]. Navitoclax significantly reduced senescence cell in *AhR+/*+ and *AhR−/−* MEFs compared to vehicle-treated controls (Figure 7A–7C). In agreement with the up regulation of AhR in wild type senescent cells (Figure 6D), Navitoclax decreased AhR receptor levels (Figure 7D), enforcing the idea of coordinated regulation between AhR expression and senescence. As expected, Navitoclax significantly reduced the expression of senescence markers *p16^Ink4a^* and *p21^Cip1^* in *AhR−/−* MEFs compared to vehicle-treated cultures (Figure 7E, 7F). Interestingly, Navitoclax increased p53 expression in AhR deficient MEFs (Figure 7G).

**Figure 8:**
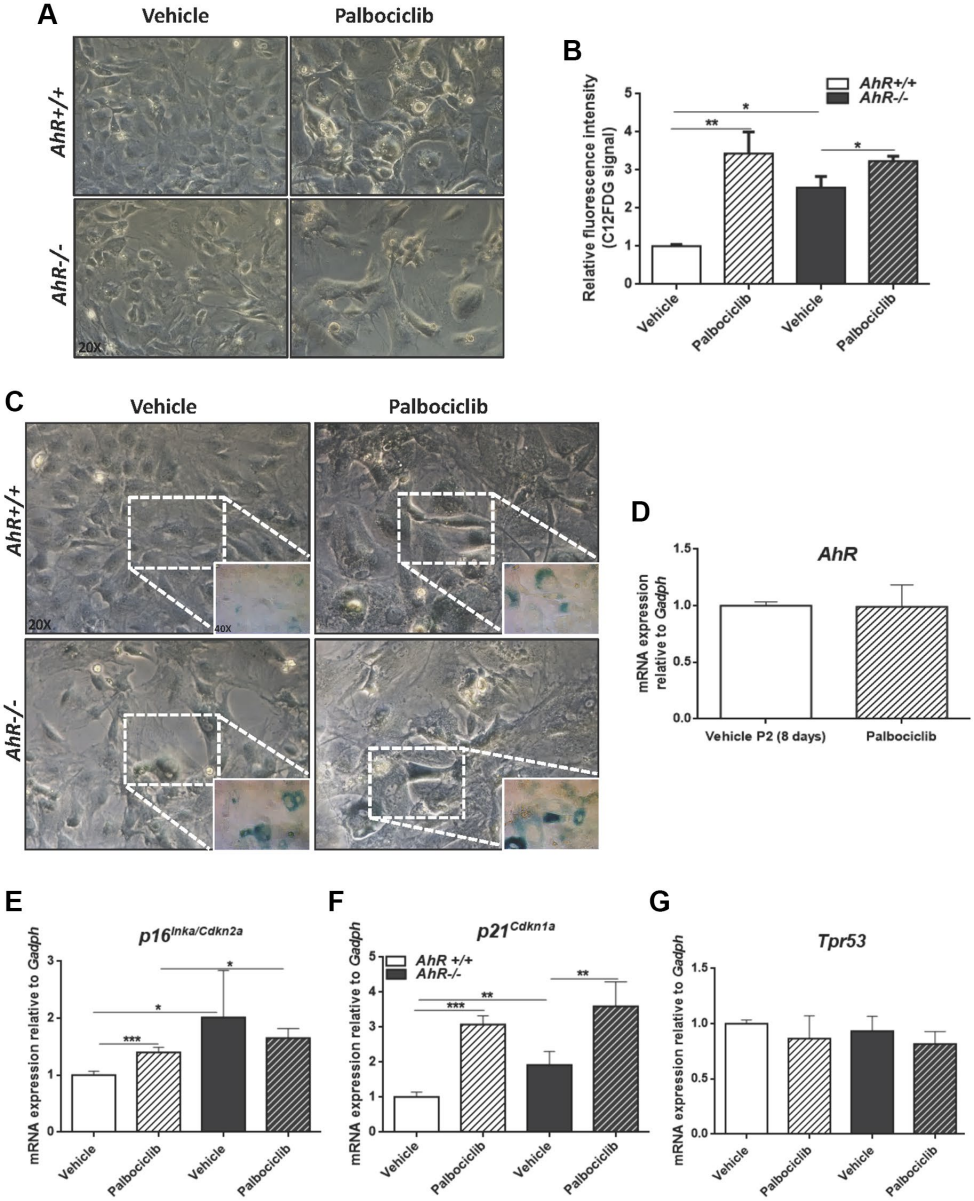
**CDK4/6 inhibitor Palbociclib induces senescence in MEF cells and mimicks AhR deficiency**. Embryonic fibroblasts were cultured for 48 h, then plated at 4 x 10^5^ cells per 10-cm plate. Two days later, cells were treated with 4 μM CDK4/6 inhibitor Palbociclib/PD-0332991 for 8 days. (**A**) Bright-field microscopy of *AhR+/+* and *AhR−/−* MEFs untreated or treated with Palbociclib. (**B**) Cell senescence measured as SA-β-Gal activity in *AhR+/+* and *AhR−/−* MEFs. SA-β-Gal activity was analyzed by FACS using the β-galactosidase fluorescent substrate C12FDG. Results are normalized to vehicle-treated wild-type MEFs. (**C**) X-gal staining in untreated and Palbociclib treated *AhR+/+* and *AhR−/−* MEFs. (**D**) *AhR* mRNA level was determined by RT-qPCR in *AhR+/+* MEFs under both experimental conditions using oligonucleotides indicated in Supplementary Table 1. Determinations were done after 8 days of treatment with Palbociclib or vehicle (control). (**E**–**G**) mRNA levels of senescence driver genes *p16Ink4a* (**E**), *p21Cip1* (**F**) and *Trp53* (**G**) was determined in *AhR+/+* and *AhR−/−* MEFs by RT-qPCR using oligonucleotides indicated in Supplementary Table 1. *Gapdh* was used to normalize target gene expression (△Ct) and 2^−△△Ct^ to calculate changes in mRNA levels with respect to wild type or untreated conditions. Data are shown as mean + SD (^*^P < 0.05; ^**^P < 0.01; ^***^P < 0.001).

The CDK4/6 inhibitor Palbociclib/PD0332991 is a cell cycle inhibitor and senescence inducer [50]. Palbociclib efficiently increased senescence in both *AhR+/+* and *AhR−/−* MEFs, with more pronounced effects in the former due to the lower basal incidence of senescent cells (Figure 8A–8C). Treatment of wild type MEFs at passage 2 with Palbociclib for 8 days did not alter AhR expression compared to control cultures (Figure 8D), perhaps because under non-proliferating confluent conditions *AhR+/+* MEFs normalize AhR expression, regardless of the levels of senescence. As expected, Palbociclib significantly increased *p16^Ink4a^* and *p21^Cip1^* expression in MEFs of both genotypes, despite less effect in *AhR−/−* MEFs, likely due to their higher basal level of both proteins (Figure 8E, 8F). p53 expression was not altered by Palbociclib in either genotype (Figure 8G).

**Figure 9:**
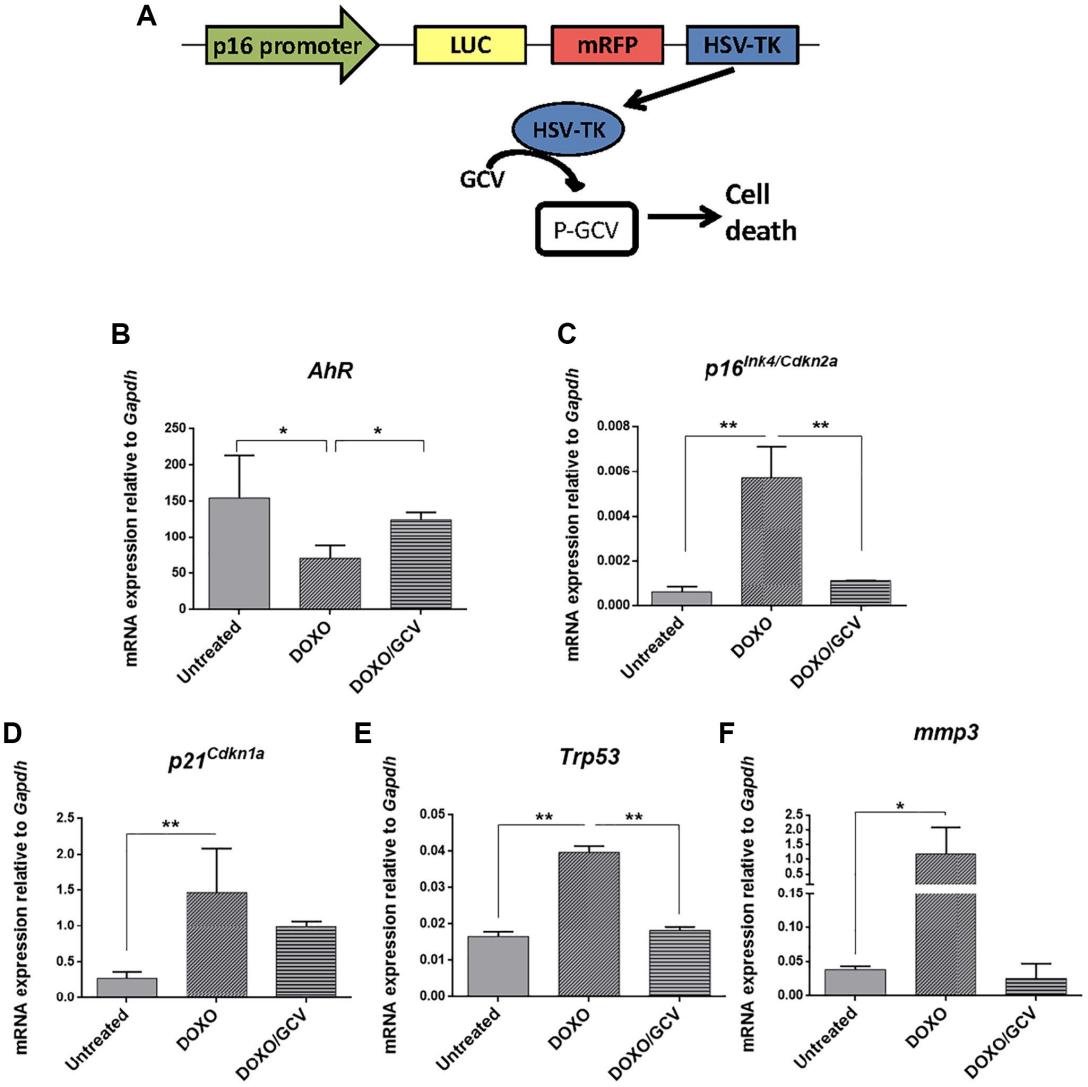
**AhR expression varies with the eradication of senescence *in vivo* in *p16^Ink4a^-3MR* transgenic mice.** (**A**) Schematic representation of the construct used to generate the *p16-3MR* transgenic mice to deplete senescent cells *in vivo* by ganciclovir treatment. *p16-3MR* mice at 4–7 months of age were treated with doxorubicin (DOXO), doxorubicin + ganciclovir (DOXO+GCV) or vehicle PBS (vehicle). mRNA expression of *AhR* (**B**)*, p16^Ink4a^* (**C**), *p21^Cip1^* (**D**), *p53* (**E**) and Mmp3 (**F**) was determined by RT-qPCR in lung tissue using the oligonucleotides indicated in Supplementary Table 1. *Gapdh* was used to normalize target gene expression (△Ct) and 2^−△△Ct^ to calculate changes in mRNA levels with respect to wild type or untreated conditions. Data are shown as mean + SD (^*^P < 0.05; ^**^P < 0.01).

The correlation between AhR expression and senescence in adult tissues was also analyzed *in vivo* using *p16^Ink4a^-3MR* transgenic mice in which senescent cells can be eliminated by apoptosis by the metabolism of ganciclovir driven by the *p16^Ink4a^* promoter [51] (Figure 9A). Senescence induction by doxorubicin reduced AhR expression in *p16^Ink4a^-3MR* mice lungs (taken as target tissue) whereas co-treatment with ganciclovir restored receptor levels (Figure 9B). Accordingly, levels of *p16^Ink4a^* and *p21^Cip1^* were upregulated by doxorubicin to be reduced after the elimination of senescent cells by ganciclovir (Figure 9C, 9D). In addition, the expression of *p53* and the SASP factor *Mmp3* was also modulated following induction or elimination of senescent cells (Figure 9E, 9F).

## DISCUSSION

Aging increases the incidence of numerous life-threatening diseases, including cardiovascular, oncogenic and neurodegenerative pathologies, which limit life expectancy. An increasing number of studies report functions of AhR in aging [52–55], having positive or negative roles, depending on species, cell type and receptor levels [3, 8, 56, 57]. On the other hand, AhR has emerged as a regulatory factor in pluripotency and stemness in different cell types, including undifferentiated human teratoma cells [5, 10, 58]. Accordingly, this receptor is required to block hepatocarcinogenesis induced by the toxin diethyl nitrosamine [1, 12], and AhR-lacking mice show increased tumor burden due to expansion of undifferentiated hepatic pluripotent cells [12] that unusually remain in the adult liver even after age-dependent maturation [13]. Notably, aging induced a significant increase in spontaneous (not induced) liver tumors in AhR-deficient mice that was accompanied by larger numbers of liver progenitor cells, eventually contributing to tumorigenesis as they stimulate hepatic regeneration [12, 59]. Since AhR expression and activation decrease with age in wild type mice, we suggest that partial deficiency in receptor levels may facilitate or account for the existence of a basal tumor load and reduction in undifferentiated cells. In addition, the remaining AhR expression could be enough to restrain liver tumorigenesis, decreasing its incidence compared to fully depleted *AhR−/−* animals.

Cellular senescence has been linked to physiological aging and age-related diseases [15, 22]. Remarkably, senescence not only increases during *in vivo* reprogramming of tumors by OKSM pluripotency factors [24], but also can induce reprogramming *in vivo* through p16^Ink4a^ [25, 33]. Thus, there is functional interaction between senescence and stemness that impacts tumor development and tissue regeneration [17, 18, 60, 61]. In agreement with that hypothesis, aging in AhR-deficient mice result in higher levels of liver senescence with up-regulation of inducers p16^Ink4a^, p21^Cip1^, IL1 and TNFα. Moreover, AhR depletion also altered hepatic levels of pleiotropic SASP factors MCP-2, MMP-12, FGF, HGF and VEGF. Since the SASP can contribute to tumor development [62] producing proinflammatory cytokines, growth factors and extracellular matrix remodelers that facilitate the expansion of transformed cells, we suggest that the SASP factors in *AhR−/−* liver could contribute to their enhanced hepatocarcinogenesis. Pluripotency and reprogramming inducers OCT4 and NANOG, both repressed in an AhR-dependent manner during liver tumorigenesis and regeneration [12], were overexpressed in AhR-null livers in parallel with their enhanced senescence and tumor burden. We therefore propose that AhR is at the crossroad of signaling pathways that integrate stemness, senescence and tumor development during liver aging. ChIP data indicate that AhR transcriptionally regulates the expression of p16^Ink4a^, p21^Cip1^ and TNFα, thus supporting a plausible causal role of this receptor in senescence.

AhR modulates senescence in different cell types and organs, including mesenchymal embryonic fibroblasts [28]. Using passaging as a model for aging in culture, we found that MEFs from *AhR−/−* mice are more susceptible to senescence as determined by higher levels of SA-β-Gal, *p16^Ink4a^* and *p21^Cip1^*. These data again suggest that AhR inhibits senescence and, in fact, its expression in *AhR+/+* MEFs with passage may reveal a cellular response to this such process. Indeed, the Bcl-2 family inhibitor Navitoclax [49] is a senolytic and eliminates senescent cells more efficiently in the absence of AhR, with downregulation of SA-β-Gal, *p16^Ink4a^* and *p21^Cip1^*. AhR levels declined in senescent *AhR+/+* MEFs, in agreement with the idea that reduced senescence correlates with receptor inhibition. Likewise, the CDK4/6 inhibitor Palbociclib/PD0332991 [50] increased senescence and, in parallel, AhR levels increased early during the senescence process. Interestingly, the effects of AhR on embryonic senescence were also seen in adult aged fibroblasts from *AhR−/−* mouse tails, in which cell cycle blockade and higher levels of SA-β-Gal, *p16^Ink4a^* and *p21^Cip1^* correlated with senescence. Further, *AhR−/−* adult fibroblasts had decreased mitochondrial activity that could result from their enhanced senescence as previously suggested [47, 63]. Finally, elimination of senescent cells *in vivo* by activation of a p16^Ink4a^ promoter-driven killer gene in *p16-3MR* transgenic mice further support our conclusion that lower senescence rates are associated to elevated AhR expression levels.

In summary, we provide here experimental evidence that the aryl hydrocarbon receptor is a molecular intermediate in signaling pathways that govern the interaction between senescence and reprogramming. AhR would serve as a limiting factor in the control of both senescence and pluripotency, eventually restricting tumor development with age. Physiological modulation of AhR activity may present a potential approach to circumvent the progression of liver tumors in humans.

## METHODS

### Mice and treatments

*AhR+/+* and *AhR−/−* mice (C57BL/6N x 129 sV) were produced by homologous recombination in embryonic stem cells as described [38, 39] and used at 4–6 weeks (young group) or 15–22 months (aged group). For AhR induction, mice were injected with 4 μg/kg 6-formylindolo[3,2-b]carbazole (FICZ) diluted in PBS. Untreated control mice were injected with the same volume of DMSO. At the end of treatments, mice were sacrificed, and livers extracted. *p16-3MR* transgenic mice were generated as reported [51]. Mice at 4–7 months of age were treated i.p., with a single dose of 10 mg/kg doxycycline and seven days later with 25 mg/kg ganciclovir once per day for 5 consecutive days to clear senescent cells. Control mice received the same volume of vehicle (PBS). Experiments using mice were performed in accordance with the National and European legislation (Spanish Royal Decree RD53/2013 and EU Directive 86/609/CEE as modified by 2003/65/CE, respectively) for the protection of animals used for research. Experiments using mice were approved by the Bioethics Committee for Animal Experimentation of the University of Extremadura (Registry 109/2014) and the Junta de Extremadura (EXP-20160506-1). Mice had free access to water and rodent chow.

### Mouse embryonic fibroblasts (MEFs)

*AhR+/+* and *AhR−/−* MEFs were isolated from 14.5 d.p.c. mouse embryos as reported [64] and chemically induced senescence was performed as indicated [65]. Briefly, MEFs cryopreserved two days after isolation were thawed and cultured for 48 h in Dulbecco’s modified Eagle’s medium (DMEM) supplemented with 10% FBS, 2 mM L-glutamine, 100 U/ml penicillin and 100 mg/ml streptomycin at 37°C and 5% CO_2_ atmosphere. Cells were then trypsinized and plated at 4 × 10^5^ cells per 10-cm culture dish. For senescence induction, cultures were treated with 4 μM of the CDK4/6 inhibitor Palbociclib/PD-0332991 (Merck, PZ-0199) for 8 days, refreshing media and drug on day 4. Treated cells were trypsinized, washed with PBS and plated in complete DMEM medium for at least 24 h before starting the experiments. For depletion of senescent cells, MEFs were treated for 48 h with 10 μM of the Bcl-2 inhibitor Navitoclax (MedChemExpress, HY-10087) as described [49]. In both treatments, equivalent volumes of solvent were used as vehicle controls.

### Adult primary fibroblasts

Adult primary fibroblasts were obtained from tail tips of *AhR+/+* and *AhR−/−* mice. Mice were sacrificed and biopsies of the last 3 cm of tail were excised, cleaned in PBS and disinfected in 70% ethanol. After drying, tissues were sectioned in small pieces and placed in 0.25% trypsin (Invitrogen) in PBS and disaggregated by shaking for 90 min at 200 rpm and 37°C. The resulting digestion was filtered through a 40 μm mesh, washing the remains with RPMI medium. The filtrate was centrifuged at 200 g for 5 min and the cell pellet homogenized in RPMI medium containing 10% FBS, 50 μM 2-mercaptoethanol, non-essential amino acids (Gibco) and 1% penicillin/streptomycin at 37°C in a 5% CO_2_ atmosphere. At that time, fibroblast cultures were considered passage 0. After 24 h, cultures were washed with PBS and fresh medium added. Thereafter, the medium was changed every other day.

### Gene expression analysis

Total RNA was isolated from *AhR+/+* and *AhR−/−* mouse livers using the Trizol reagent (Life Technologies). Following centrifugation, supernatants were precipitated with isopropanol, centrifuged again and pellets dissolved in DEPC-treated water. High Pure RNA Isolation Kit (Roche) was used to further purified RNA from tissues and *AhR+/+* and *AhR−/−* MEFs. To analyze mRNA levels by RT-qPCR, reverse transcription was performed using random priming and the iScript Reverse Transcription Super Mix (Bio-Rad). Real-time PCR was used to quantify mRNA levels of *AhR*, *p16^Ink4a^, p21^Cip1^, IL1, TNFα, Tpr53, Mmp3, Cyp1a1* and *Gapdh* using SYBR^®^ Select Master Mix (Life Technologies) in a Step One Thermal Cycler (Applied Biosystems) as indicated [5]. *Gadph* was used to normalize gene expression (△Ct) and 2^−△△Ct^ to calculate changes in RNA levels with respect to controls. Primer sequences used are indicated in Supplementary Table 1.

### Immunoblotting

SDS–PAGE and immunoblotting were performed using total protein extracts obtained from *AhR+/+* and *AhR*−/− mouse livers as described [13]. Briefly, tissues were extracted and homogenized in ice-cold lysis buffer; following centrifugation, protein concentration was determined in the supernatants using the Coomassie Plus protein assay reagent (Pierce) and bovine serum albumin (BSA) as a standard. Aliquots of 20–30 μg protein were electrophoresed in 8% SDS-PAGE gels, which were transferred to nitrocellulose membranes. After blocking in a TBS-T (50 mM Tris-HC1 pH 7.5, 10 mM NaCl, 0.5% Tween 20) containing 5% non-fat milk, blots were sequentially incubated with the primary antibodies anti-AhR (Enzo 1:1000), anti-p53 (Cell signaling 1:1000) and anti-β-Actin (Sigma 1:1000) and secondary antibodies, washed in TBS-T and developed using the Super-signal luminol substrate (Pierce). Blots were scanned and protein expression quantified using ChemiDoc XRS+ equipment (Bio-Rad).

### Senescence-associated β-galactosidase activity

Senescence-associated β-galactosidase activity (SA-β-gal) was analyzed in primary fibroblasts, isolated liver cells and liver tissue following published protocols [66, 67]. For primary adult fibroblasts and MEFs, the β-galactosidase fluorescent substrate C12FDG was added to the cultures at 60 μM for 20 min at 37°C with gently shaking. C12FDG positive cells were quantified by flow cytometry using a Cytoflex S cytometer (Beckman Coulter) when analyzed in suspension, or an Olympus FV1000 confocal microscope and FV10 software (Olympus) when analyzed in attached cultures. In some experiments, MEFs were stained with X-Gal as substrate to visualize senescence by bright-field microscopy in a Olympus BX51 microscope. Isolated liver cells were obtained by mechanical disaggregation of liver tissue in a gentleMACS Octo Dissociator (Miltenyi). The resulting cell suspension was filtered through 30 μm mesh and centrifuged for 5 min at 1800 g. Cell pellets were resuspended in PBS and incubated with the C12FDG substrate as indicated above. Cells were then resuspended in 0.1% BSA containing 10 μg/μl Hoechst 33258 and analyzed for senescence in a Cytoflex S cytometer (Beckman Coulter). For liver tissue, once fixed in buffered formaldehyde and embedded in paraffin, 3–5 μm liver sections were deparaffinized, rehydrated and stained for SA-β-Gal activity using X-Gal as described [67]. Sections were observed and photographed using an Olympus BX51 microscope.

### Hematoxylin/eosin staining of liver sections

Mouse livers were processed for H&E staining as described [13]. Tissues were fixed overnight at room temperature in buffered formalin and embedded in paraffin. 3 μm sections were deparaffinated, rehydrated and incubated for 3 min with hematoxylin; after washing with tap water, they were stained with eosin for 1 min. Sections were then dehydrated, mounted and observed in a NIKON TE2000U microscope using 4× (0.10 numeric aperture), 10× (0.25 numeric aperture) an 20× (0.40 numeric aperture) objectives.

### Immunofluorescence

Liver sections were manually deparaffinated and rehydrated in PBS. Antigen unmasking was performed in citrate buffer at pH 6. After washing in PBS containing 0.05% Triton X-100 (PBS-T), non-specific epitopes were blocked by incubation for 1 h at room temperature in PBS-T containing 0.2% gelatin and 3% BSA (PBS-T-G-B). Sections were incubated overnight at 4°C with the following primary antibodies diluted in PBS-T-G-B: anti-p16^Ink4a^ (Santa Cruz Biotechnology 1:100), anti-p21^Cip1^ (Abcam, 1:100), anti-α-SMA (Sigma 1:200) anti-OCT4 (Santa Cruz Biotechnology 1:200) and anti-NANOG (Novus Biologicals 1:100). Following washing in PBS-T, sections were incubated for 1 h at room temperature with Alexa-488, Alexa-550 or Alexa-633 labeled secondary antibodies diluted in PBS-T-G-B. After additional washing, sections were dehydrated and mounted in PBS:glycerol (1:1). Primary fibroblasts on glass coverslips were washed with PBS and fixed for 10–15 min at room temperature with 3.5% paraformaldehyde in PBS. After washing, cells were permeabilized by PBS containing 0.1% Triton X-100 and 0.2% BSA, washed in PBS and blocked for 30 min in 2% BSA in PBS. Primary antibodies anti-p16^Ink4a^ (Novus Biologicals 1:200), anti-p21^Cip1^ (Santa Cruz Biotechnology 1:100) and anti-Cyclin E (Santa Cruz Biotechnology 1:75) were added in blocking solution overnight at 4°C. Following three washes with PBS, secondary antibodies labeled with Alexa-488 or Alexa-633 were added for 1 h. Liver and fibroblast samples were visualized using an Olympus FV1000 confocal microscope (Olympus). Fluorescence analysis was done using the FV10 software (Olympus). DAPI was used to stain cell nuclei.

### Mitochondrial membrane potential

Tail tip fibroblasts were seeded in 96-well plates and the mitochondrial membrane potential (MMP) determined using the mitochondrial membrane potential kit (Sigma) following the manufacturer’s recommendations. The red/green fluorescence intensity ratio was used to determine MMP values. To determine mitochondrial activity, fibroblasts were stained with 4 nM tetramethylrodamine for 30 min in complete RPMI medium without phenol red at 37°C and 5% CO_2_. Samples were analyzed using an Olympus FV1000 confocal microscope and FV10 software (Olympus). Additional determination of MMP was done using the JC-10 dye. Fibroblasts were processed as indicated above and incubated with JC-10 for 30 min at 37°C and 5% CO_2._ Samples were analyzed using a Cytoflex S flow cytometer (Beckman Coulter).

### Chromatin immunoprecipitation (ChIP)

Liver tissue samples (60 mg) were finely minced, and DNA-protein interactions stabilized by incubation in 1% formaldehyde for 15 min at room temperature with agitation. Incubation was stopped by adding 0.125 glycine for 5 min. After washing in ice-cold PBS, samples were homogenized, and cell suspensions incubated for 10 min at 4°C in SDS lysis buffer containing protease inhibitors (Complete protease inhibitor cocktail, Roche). The remaining procedures were performed as described [68]. Positive controls were input DNAs and negative controls were an absence of antibodies. Real-time PCR (qPCR) was carried out using IQ-SYBR Green in a Step One Thermal Cycler (Applied Biosystems). Primers are indicated in Supplementary Table 2. Data are presented as percentage of DNA input in the antibody-containing immunoprecipitants minus the percentage of DNA input in the corresponding negative controls.

### Senescence associated secretory phenotype (SASP)

Liver tissue was homogenized in lysis buffer (Bio-Rad) supplemented with protease inhibitors (Complete protease inhibitor cocktail, Roche) using a ratio of 1 mg tissue per 4 μl buffer. Samples were centrifuged at 500 g for 15 min and protein concentration determined in the supernatants. They were diluted with Bioplex sample diluent to a final concentration of 300 μg/ml. Plasma was obtained from whole blood taken from the left ventricle by cardiac puncture. Aliquots of 400–500 μl of blood were aspirated into a 2 ml syringe containing anticoagulant and immediately placed in EDTA containing tubes. Blood cells were separated by centrifugation at 1500 g for 15 min and the resulting plasma diluted 1:4 in Bioplex sample diluent. Cytokine content was analyzed using the Bio-Plex Multiplex immunoassay kit following the manufacturer's instructions.

### Hexokinase activity assay

To determine glucose uptake by the liver, the Picoprobe Hexokinase activity kit (Biovision) was used following the manufacturer's instructions. 30 mg of tissue were homogenized in 300 μl cold HK assay buffer. After centrifugation for 5 min at 10.000 g and 4°C, supernatants were collected and used following the provider’s instructions.

### Quantification of liver progenitor cells

Livers were removed and processed using the gentleMACS Octo Dissociator kit (Miltenyi) to obtain homogeneous cell suspensions. Cell pellets were resuspended in 0.1% BSA in PBS and antibodies for undifferentiated liver cell markers CD133-PE (0.01 μg/μl) and CD326-EpCAM (0.3 μg/sample). A negative control was without antibodies. Cell suspensions were stirred for 20 min at 37°C and analyzed using a Cytoflex S Cytometer (Beckman Coulter).

### Statistical analyses

Quantitative data are shown as mean ± SD. Comparisons between experimental conditions was done using GraphPad Prism 6.0 software (GraphPad). The student’s *t* test was used to analyze differences between two experimental groups and ANOVA for the analyses of three or more groups. The Mann-Whitney non-parametric statistical method was used to compare rank variations between independent groups (^*^P < 0.05; ^**^P < 0.01, ^***^P < 0.001), ^****^P < 0.0001).

## Supplementary Materials

Supplementary Figure 1

Supplementary Tables
