# Supplementary material for: Aryl hydrocarbon receptor blocks aging-induced senescence in the liver and fibroblast cells
Source: Aging (Albany NY). 2022 May 26;14(10):4281–304. doi: 10.18632/aging.204103 (PMC9186759; doi:10.18632/aging.204103)
Supplement: Supplementary Figure 1 [file aging-14-204103-s001.pdf]

SUPPLEMENTARY FIGURE

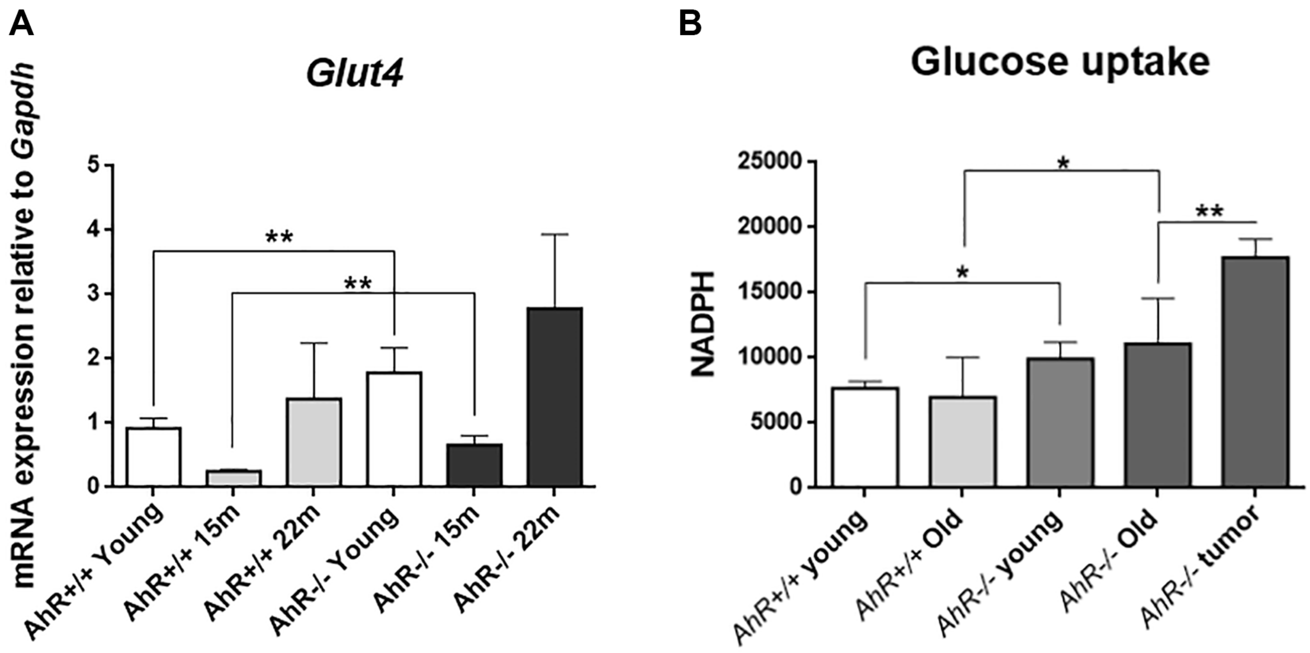

Supplementary Figure 1. (**A**) mRNA level of the glucose transporter *Glut4* was determined by RT-qPCR in *AhR+/+* and *AhR−/−* livers at the indicated ages using the oligonucleotides indicated in Supplementary Table 1. *Gapdh* was used to normalize target gene expression (△Ct) and $2^{-\triangle\triangle Ct}$ to calculate changes in mRNA levels with respect to wild type or untreated conditions. (**B**) Glucose uptake was measured in young and aged *AhR+/+* and *AhR−/−* livers by the Picoprobe Hexokinase activity assay kit (Biovision). Data are shown as mean + SD. ($^{*}P < 0.05$; $^{**}P < 0.01$).
