# Supplementary material for: Aryl hydrocarbon receptor blocks aging-induced senescence in the liver and fibroblast cells
Source: Aging (Albany NY). 2022 May 26;14(10):4281–304. doi: 10.18632/aging.204103 (PMC9186759; doi:10.18632/aging.204103)
Supplement: Supplementary Tables [file aging-14-204103-s002.pdf]

SUPPLEMENTARY TABLES

Supplementary Table 1. Oligonucleotide sequences used for mRNA level analyses.

Gene name	Sequence 5′–3′
AhR	Fw: AGCCGGTGCAGAAAACAGTAA<br>Rv: AGGCGGTCTAACTCTGTGTGT
Cyp1a1	Fw: ACAGACAGCCTCATTGAGCA<br>Rv:GGCTCCACGAGATAGCAGTT
Tnfα	Fw: CCACCACGCTCTTCTGTCTA<br>Rv: CTCCACTTGGTGGTTTGCTA
IL1	Fw: CGGGTGACAGTATCAGCAAC<br>Rv: GACAAACTTCTGCCTGACGA
Glut4	Fw: ATGACCAAGCCCTGAATCTG<br>Rv: CGTAGGATGTGGTGATGACG
p16	Fw: TACCCCGATTCAGGTGAT<br>Rv: TTGAGCAGAAGAGCTGCTACGT<br>Fw: GTCGCAGGTTCTTGGTCACT<br>Rv: CGAATCTGCACCGTAGTTGA
p21	Fw: GCCTTAGCCCTCACTCTGTG<br>Rv: AGCTGGCCTTAGAGGTGACA
p53	Fw: TGGAAGACTCCAGTGGGAA<br>Rv: TCTTCTGTACGGCGGTCTCT
Mmp3	Fw: AGTCAGGGTCACCCACAAAG<br>Rv: GCATTGGGTATCCATCCATC
Gapdh	Fw: TGAAGCAGGCATCTCAGGG<br>Rv: CGAAGGTGCAAGAGTGGGA

Supplementary Table 2. Oligonucleotide sequences used for chromatin immunoprecipitation (ChIP).

Gene name	Sequence 5′–3′
p16	Fw: GGCACTCCCAGCAAGTAGAT<br>Rv: CACAAACGTGCCTCCTATACA
p21	Fw: TTTGTTGTCCTCGCCCTCAT<br>Rv: ACGCACGTACACAGACACA
Tnfα	Fw: CCAGACACTCACCTCATCCC<br>Rv: TGGAACTGGCAGAAGAGGC
Gapdh	Fw: TGAAGCAGGCATCTCAGGG<br>Rv: CGAAGGTGCAAGAGTGGGA
